# Emergency management: vitreous loss

**Published:** 2018-11-09

**Authors:** William Dean

**Affiliations:** 1 Clinical Research fellow: London School of Hygiene and Tropical Medicine, London, UK.

**The most common mistake when managing vitreous loss is to leave some of it behind in the anterior chamber or up to the wound. Knowing when to stop using the vitrector, and how to tell whether there is any vitreous remaining, is crucial.**

**Figure:**
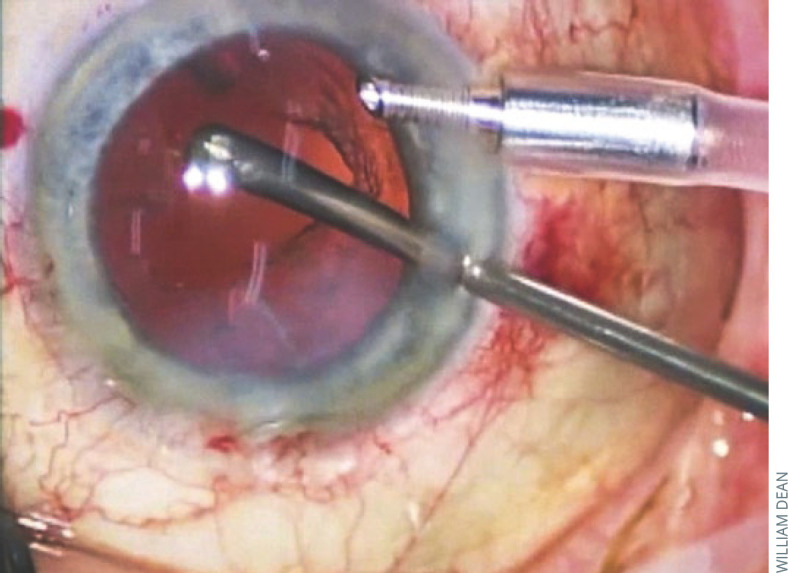
Vitrectomy after a posterior capsular tear with vitreous loss.

A posterior capsular tear (PCT) with vitreous loss is an emergency that can occur at different stages of a cataract operation.

**Signs** include sudden deepening of the anterior chamber, momentary dilation of the pupil, visible vitreous, a visible PCT, a peripheral clear red reflex, and/or excess movement of the nucleus.

All cataract surgeons need to know how to manage vitreous loss safely. It is never acceptable to leave vitreous in the anterior chamber, especially if it extends to the main wound or paracentesis.

**Differential diagnoses** may include a simple fold in the posterior capsule, which may appear like the edge of a PCT. Soft lens matter may mimic the peripheral clear red reflex of a PCT.

## Remedies and immediate management

Once vitreous loss has been diagnosed, it must be effectively managed. Do not panic. Stop any aspiration, calmly assess the situation, then gently remove the instruments from the eye. If possible, inject dispersive viscoelastic into the anterior chamber before removing the instruments.

## Step 1: Release the pressure on the eye

- Make a superior rectus suture
- Consider ‘lifting’ the speculum forwards
- Reduce the pressure of irrigation and aspiration (Simcoe or automated) when re-inserted into eye
- Reduce the height of the irrigation fluid bag
- Do not exert pressure on the globe with scleral fixation or other forceps.

**Figure 1:**
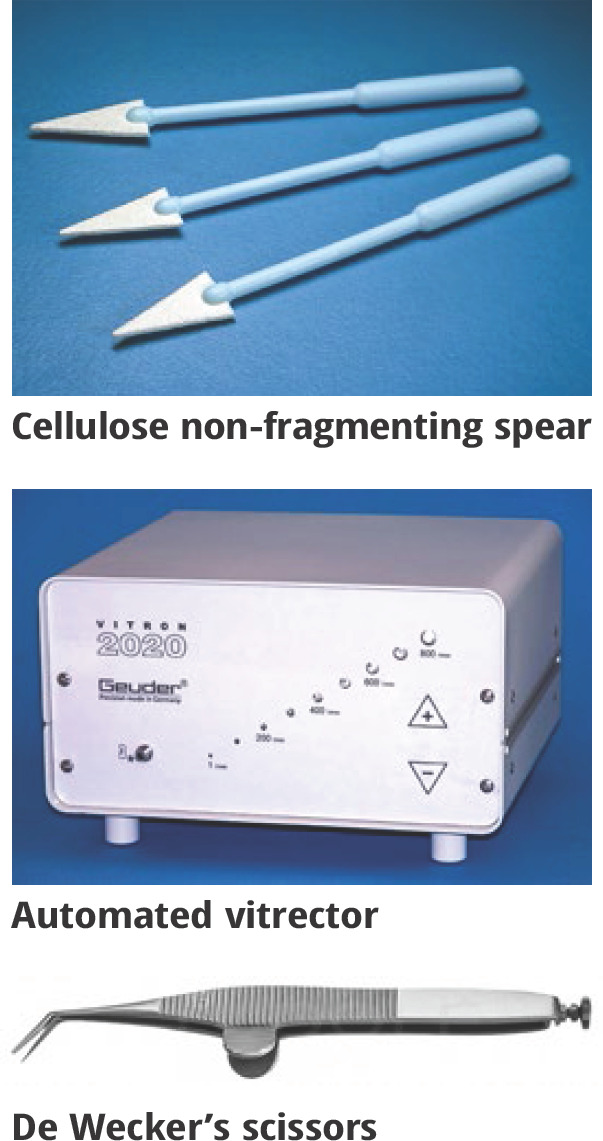
Instruments used to manage vitreous loss

## Step 2: Remove the vitreous

When using an automated vitrector, keep the **aspiration (vacuum) rate low** and the **cut rate high**. Keep the **irrigation (infusion) rate low**. Vitreous is cut and removed at the site of the PCT, around the pupil margin, and in the anterior chamber. Perform a limited anterior vitrectomy through the PCT. Stop using the vitrector when you are confident there is no further vitreous in the anterior chamber.

A manual sponge vitrectomy is performed using a cellulose non-fragmenting spear (Figure 1). Depress this on the posterior wound to express vitreous, and then cut the vitreous using sharp intraocular scissors such as De Wecker's or curved Westcott's. Repeat until there are no more strands of vitreous coming to the wound and the sponge.

Triamcinolone 40 mg/ml stains the vitreous strands white when injected into the anterior chamber, which makes it much easier to do a complete vitrectomy. A simple technique is to use a viscoelastic cannula to sweep just above the iris, underneath the wounds. If there are strands of vitreous, the cannula will catch them and the pupil will peak. These strands need to be cut.

Remove all vitreous from the anterior chamber, especially from wounds: don't leave an oval pupil. Preserve as much of the lens capsule as possible. After vitrectomy, only insert a posterior chamber IOL if you are sure there is sufficient capsule to hold it in place.

## Step 3: Check that all the vitreous has been removed

Make final checks to identify any vitreous remaining in the anterior chamber and any strands of vitreous extending to wounds. Acetlycholine 20 mg/ml solution or preservative-free pilocarpine (2% or 4% drop diluted in 1 ml normal saline) may be injected into the anterior chamber after posterior chamber IOL insertion. This causes pupil constriction, so if there are any strands of vitreous remaining these will cause a ‘peaked’ pupil.

## Step 4: Referral

If a posterior-capsule tear occurs and any part of the cataract nucleus drops into the vitreous, then urgent referral to a vitreo-retinal specialist is needed. Never try to retrieve nucleus fragments from the vitreous, as this will damage the lens capsule, iris, and cornea, and make surgery much more difficult in future.

## How to prepare for this emergency

With the theatre team, practise setting up the vitrector, if one is available. Do this until everyone feels competent.

If you have access to a wet lab or surgical skills centre, practise using animal eyes or by injecting egg white into the posterior chamber of an artificial eye and causing a PCT with a needle. Effective management of vitreous loss should be practised away from the patient and the stress of a live complication.

